# Antibacterial Activity and Action Mode of Lactobionic Acid Against *Cronobacter sakazakii*: With Insights into Cell Wall, Membrane, and Macromolecule Targeting

**DOI:** 10.3390/foods15030535

**Published:** 2026-02-03

**Authors:** Shimo Kang, Siyuan Wang, Shuqi Shen, Yaqi Zhang, Na Liu, Xiqing Yue

**Affiliations:** 1 Shanghai Institute of Nutrition and Health, University of Chinese Academy of Sciences, Chinese Academy of Sciences, Shanghai 200031, China; shimokang@sinh.ac.cn (S.K.);; 2 School of Food Science and Technology, Jiangnan University, Wuxi 214122, China; 3 College of Food Science, Shenyang Agricultural University, Shenyang 110161, China

**Keywords:** lactobionic acid, *Cronobacter sakazakii*, antibacterial activity, action mode

## Abstract

Lactobionic acid (LBA) has demonstrated antibacterial activities against multiple foodborne bacteria; however, few studies have reported on its effect against *Cronobacter sakazakii*. In this study, the antibacterial activity and mode of LBA against *C. sakazakii* were explored. The minimum inhibitory concentration (MIC) and minimum bactericidal concentration (MBC) of LBA against *C. sakazakii* were 12.5 and 25 mg/mL, respectively. LBA exhibited bacteriostatic activity at sub-MIC and bactericidal activity at concentrations ≥ MIC. Alkaline phosphatase (AKP) activity, cell outer membrane (OM) permeability, protein leakage, and gel electrophoresis results suggested that LBA increased the permeability of the cell wall and OM, leading to intracellular protein leakage and a decrease in protein contents and activity, indicating LBA damage to the cell wall and membrane. Among these, the rapid AKP activity surge reached 4.37 U/gprot at 2 MIC, and the OM permeability dramatically increased up to 10 min and stabilized after 30 min. Microscopic observations confirm the disruption to the cell wall and membrane, further showing that LBA disrupted the integrity of the cell wall and membrane. Moreover, LBA disturbs normal cellular functions by binding to deoxyribonucleic acid (DNA), as reflected by the competitive binding assay. Overall, LBA possesses potential multiple applications in the food industry due to its natural and antibacterial properties.

## 1. Introduction

*Cronobacter sakazakii* is an opportunistic foodborne pathogen that can tolerate extremely dry environments, thereby surviving and persisting in various low-moisture food products [1,2]. Such products include flour, powdered milk, powdered infant formula (PIF), herbal teas, starches, spices, etc. [3,4]. Notably, PIFs are often contaminated by *C. sakazakii*, and the most recent food safety recall of PIF was in 2022 [5]. Hence, infants and the elderly with weakened immune systems are more susceptible to *C. sakazakii* infections. Once infected, it is easy to develop meningitis, septicemia, and necrotizing enterocolitis, with a mortality rate of about 40~80% [6]. Additionally, *C. sakazakii* exhibits a strong ability to form biofilms that can tightly adhere to the surfaces of food equipment, packaging materials, and tableware, which reduces the efficacy of chemical disinfectants, such as hydrogen peroxide and chlorine-containing disinfectants, and even induces resistant strains to chemical disinfectants [7]. *C. sakazakii* isolates are also resistant to multiple antibiotics, such as ampicillin, cefazolin, and penicillin, and this resistance is increasing rapidly [8,9]. In contrast, natural antibacterial agents are characterized by easy accessibility, safety, and nontoxicity, with low potential for resistance development, minimal harm to humans, and sustained antibacterial efficacy, gaining extensive attention [10,11].

Lactobionic acid (LBA) is a natural organic acid that is primarily derived from the oxidation of lactose by fungal or bacterial enzymes (e.g., from *Aspergillus* or *Pseudomonas* species) and is naturally present in fermented dairy products like yogurt and kefir [12]. Numerous studies reported that LBA has exerted antioxidant, anti-obesity, prebiotic, and antibacterial effects [13,14]. Nowadays, LBA is approved to be used as an additive, gelling agent, antioxidant, and stabilizer in dessert products by the Food and Drug Administration (FDA) [13]. Regarding the antibacterial properties of LBA, Chen and Zhong (2017) first reported its antimicrobial activity against *Listeria monocytogenes* and *Escherichia coli* [15]. Subsequently, we conducted in-depth studies on the inhibitory effect and mechanism of LBA against *Staphylococcus aureus* and its biofilm formation [16,17,18,19]. LBA also exhibited antibacterial effects on *Pseudomonas fluorescens*, *Vibrio parahaemolyticus*, and *Salmonella* spp., as reported in the following studies [20,21]. Further, we indicated that LBA can serve as a food-grade additive to extend the shelf life of pasteurized milk and improve its sensory quality, implying that LBA exhibits the potential to be applied as a natural antibacterial agent [16]. However, little is known on the inhibitory effect of LBA against *C. sakazakii*.

Hence, in this study, we explore the antibacterial activity and mechanism of LBA against *C. sakazakii* from three aspects: cell wall, cell membrane, and biomolecules. The finding will expedite LBA as an antibacterial agent with potential application in preventing and controlling *C. sakazakii*.

## 2. Materials and Methods

### 2.1. Chemicals, Bacterial Strain, and Culture Condition

LBA was purchased from Sigma-Aldrich Corp. (St. Louis, MO, USA). All other chemicals and reagents were of analytical grade. The *C. sakazakii* ATCC 29544 used in this study was obtained from the American Type Culture Collection (ATCC) and isolated from the child’s throat. The strain was stored in our laboratory at −80 °C before use and transferred twice in Luria–Bertani (LB) broth before testing.

### 2.2. Minimum Inhibitory Concentration and Minimum Bactericidal Concentration

The minimum inhibitory concentration (MIC) and minimum bactericidal concentration (MBC) were determined using the method we reported previously [16]. LBA was subjected to two-fold serial dilution in Luria–Bertani (LB) broth within 96-well plates, with 100 μL of the dilution added to each well. Then, 100 μL of a bacterial culture in the logarithmic phase was added to each well. Following 24 h of incubation at 37 °C with agitation, the MIC was defined as the lowest concentration that completely inhibited visible growth. To determine the MBC, 100 μL aliquots of wells in which no bacterial growth was observed were plated onto LB agar and incubated at 37 °C for 48 h.

### 2.3. Growth Curve

The growth curve was evaluated as outlined in our earlier work [16]. Exponential-phase cells were collected via centrifugation (5000× *g*, 10 min), washed with PBS, and resuspended in fresh LB broth. Then, 100 μL of bacterial culture and 100 μL of LBA at 1/2×, 1×, and 2× MIC were added to a 96-well plate, respectively, and incubated at 37 °C. The growth curves were determined at 600 nm, taken at 2 h intervals for 24 h using a Tecan Infinite M200 Pro NanoQuant Microtiter Plate Reader (Tecan, Crailsheim, Germany).

### 2.4. Alkaline Phosphatase Activity

The permeability of the cell wall of *C. sakazakii* cells exposed to LBA was investigated by determining the activity of alkaline phosphatase (AKP) according to our previous study [16]. Exponential-phase cells were collected via centrifugation (5000× *g*, 10 min), washed, and resuspended in PBS. The suspensions were exposed to LBA at 1/2×, 1×, and 2× MIC, respectively, followed by incubation at 37 °C for varying durations. Collect the supernatant after centrifuging the cultures. The activity of AKP released into the supernatant was quantified with a commercial assay kit (Nanjing Jiancheng Bioengineering Institute, Nanjing, China) according to the manufacturer’s protocol, using a microplate reader for measurement at 520 nm wavelength.

### 2.5. Outer Membrane Permeability Analysis

The outer membrane (OM) permeability was determined by the 1-N-phenylnaphthylamine (NPN) assay as detailed in our prior research [20]. The cell suspensions were harvested, as shown above (Section 2.5), and the density was adjusted to an OD_600_ of 0.4 with PBS buffer. 20 μL of 1 mM NPN was added into a quartz cuvette with 1.5 mL bacterial suspensions, then LBA at 1/2×, 1×, 2× MIC, and PBS buffer (control) were mixed with them equally, respectively. The fluorescence intensity was performed immediately on a Hitachi F-4600 spectrophotometer (λ_ex_/λ_em_ = 350/429 nm) and monitored continuously until the signal reached a plateau.

### 2.6. Leakage of Protein

The cell suspensions were harvested, as mentioned earlier (Section 2.5). The suspensions were mixed with LBA at 1/2×, 1×, and 2× MIC, respectively, followed by incubation at 37 °C for varying durations. After incubation, the cultures were centrifuged, and the supernatant was collected. Then, determined by using the micro-protein assay after being filtered through a 0.22 μm membrane, as described in our earlier report [16].

### 2.7. Sodium Dodecyl Sulfate-Polyacrylamide Gel Electrophoresis Analysis

The cell suspensions were harvested, as stated before (Section 2.4), and treated with LBA at 1/2× and 1× MIC, followed by incubation at 37 °C for 20 h. Then, collect the pellets after centrifuging (5000× *g*, 10 min). The sodium dodecyl sulfate-polyacrylamide gel electrophoresis (SDS-PAGE) of the pellets was analyzed by a 5% stacking gel and a 10% separating gel, followed by Coomassie brilliant blue staining, as detailed in our prior research [16].

### 2.8. Microscopic Visualization

The changes in cells in morphology and ultrastructure were observed by scanning electron microscopy (SEM) and transmission electron microscopy (TEM) [22]. For SEM, the suspensions were harvested, as shown above (Section 2.4), and treated with LBA at 1/2× and 1× MIC for 2 h at 37 °C and washed with PBS, then fixed with 2.5% glutaraldehyde and dehydrated through a graded ethanol. After critical-point drying and gold spraying, images were photographed by SEM (Zeiss EVO-LS10, Cambridge, UK).

For TEM, the same pretreatment procedure as applied for SEM was employed. Samples were fixed with 2.5% glutaraldehyde and post-fixed with 1% osmic acid, then, dehydrated through a graded ethanol, permeated with white resin, and the embedded blocks were sectioned into ultrathin slices using a EM FC6 (Leica Microsystems, Wetzlar, Germany) ultramicrotome for observation under a TEM (HT7700, Hitachi High-Tech, Tokyo, Japan).

### 2.9. The Interaction of LBA to Deoxyribonucleic Acid (DNA)

#### 2.9.1. Genomic DNA Extraction

Genomic DNA extraction from *C. sakazakii* cells was performed using the TIANamp Bacteria DNA Kit (Tiangen Biotech, Beijing, China). The concentration and purity of DNA were subsequently quantified on a NanoDrop ONE spectrophotometer (Thermo Scientific, Waltham, MA, USA), based on the absorbance at 260 nm (A_260_) and the A_260_/A_280_ ratio, with acceptable purity defined as a ratio between 1.8 and 2.0.

#### 2.9.2. Fluorescence Spectroscopy

To evaluate the interaction of LBA with *C. sakazakii* genomic DNA, competitive binding experiments were conducted based on a previously established method [16]. Ethidium Bromide (EB) was mixed with 60 μg/mL DNA solution to achieve a final concentration of 2 μg/mL and incubated (37 °C, dark, 30 min). Then, the EB-DNA complex was challenged with equal volumes of LBA at 0, 1/2×, 1×, and 2× MIC and incubated 30 min under identical conditions. Fluorescence emission spectra (550~750 nm, λ_ex_ = 530 nm) were acquired using an F-4600 spectrophotometer (Hitachi, Tokyo, Japan).

### 2.10. Statistical Analysis

Experiments were conducted in triplicate. Data were analyzed with variance and Duncan’s test in SPSS software (version 22) and presented as the mean ± SDs (*n* = 3). *p* < 0.05 was considered statistically significant.

## 3. Results and Discussion

### 3.1. Evaluated Antibacterial Activity

This study first demonstrated that LBA possesses antibacterial activities against *C. sakazakii*. The MIC and MBC were 12.5 mg/mL and 25 mg/mL, respectively, confirming its superior antibacterial and bactericidal properties. The MBC for *C. sakazakii* was twice that of the MIC value. Compared with our previous study, the MIC of LBA for *C. sakazakii* was the same as that for *P. fluorescens* and 1.5 times lower than that for Methicillin-resistant *Staphylococcus aureus* (MRSA); the MBC of LBA for *C. sakazakii* was the same as that for *P. fluorescens* and 2 times lower than that for MRSA [16]. Additionally, according to other relevant studies, the MICs and MBCs of LBA for *V. parahaemolyticus*, *Shewanella baltica*, and *Shewanella putrefaciens* were lower than those of *C. sakazakii*; the MIC and MBC of the former were both 4 mg/mL, and those of the latter two were both 8 mg/mL and 16 mg/mL [21,23]. The above results suggest that LBA has a broad-spectrum antibacterial activity. The notable discrepancies in MICs and MBCs among Gram-negative bacteria are inseparable from factors such as inconsistent measurement techniques and variations in the purity of LBA from different manufacturers.

**Figure 1:**
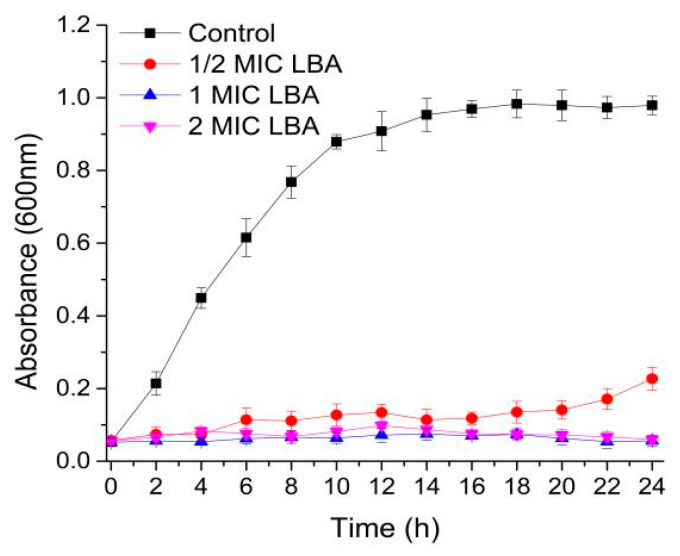
Growth curve of *C. sakazakii* after LBA treatment.

To evaluate the antibacterial activity of LBA against *C. sakazakii*, the growth curve of *C. sakazakii* after treatment with different concentrations of LBA was plotted. *C. sakazakii* without LBA treated exhibited rapid growth, and showed a maintained stabilization after incubating for 18 h (Figure 1). While the growth of *C. sakazakii* was completely inhibited after treatment with LBA at 1 and 2 MIC, demonstrating its potent bactericidal activity. This inhibition effect was concentration-dependent, as evidenced by the reduced efficacy observed at the 1/2 MIC concentration. The results suggested that LBA showed superior bactericidal and bacteriostatic properties against *C. sakazakii* in a dose-dependent manner. Therefore, LBA has potential as an effective natural antibacterial agent, and thus, its specific antibacterial mechanisms merit further elucidation.

### 3.2. Changes in Cell Wall Permeability

Alkaline phosphatase (AKP) is an intracellular enzyme that is found between the bacterial cell wall and cell membrane. Once the cell wall was damaged, AKP would leak into the extracellular space, leading to an increase in AKP activity in that area [24]. Therefore, detecting alterations in extracellular AKP activity levels can reflect the permeability of the cell wall.

**Figure 2:**
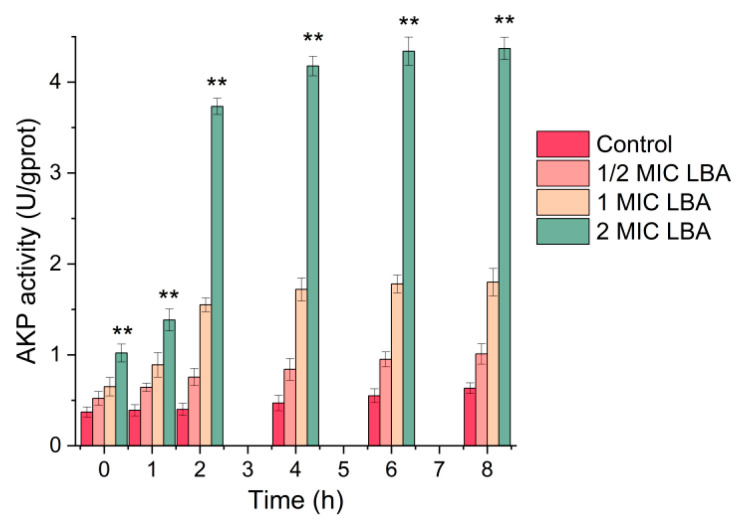
Changes in the AKP activity of *C. sakazakii* after LBA treatment. ‘**’, *p* < 0.01.

As shown in Figure 2, the AKP activity of *C. sakazakii* without LBA treatment exhibited no obvious change, which maintains the average AKP activity of *C. sakazakii* at 0.377 U/gprot. However, AKP activity increased with increasing LBA concentration and extended treatment time when *C. sakazaki* cells were subjected to different LBA concentrations for varying periods. Notably, exposure of *C. sakazaki* to LBA resulted in a rapid increase in AKP activity within 2 h, and the maximum level of AKP activity (4.37 U/gprot) was reached after 8 h of treatment with 2 MIC LBA (Figure 2). These findings indicated that LBA exposure resulted in a rapid leakage of AKP, as well as an increase of cell wall permeability, suggesting damage to the cell wall of *C. sakazakii*. The results were consistent with our previous studies that LBA can disrupt the integrity of the cell wall of *P. fluorescens* and MRSA by quickly increasing the AKP activity within 2 h [16]; they are superior to the results of black pepper petroleum ether extract, which required 4 h to elevate AKP activity in *Salmonella enterica* serovar Typhimurium (*S. typhimurium*) and *L. monocytogenes*. [25]. Additionally, Fei et al. (2025) found that *Lonicera japonica Thunb.* polyphenols damage the cell wall of *C. sakazakii* by inducing AKP leakage [22].

### 3.3. Changes in Cell Outer Membrane Permeability

Gram-negative bacteria’s cell envelopes are known to have an outer membrane (OM) and inner membrane, which function as an efficient permeability barrier to keep out hydrophobic substances and macromolecules [26]. Compared to G^+^ bacteria, the unique OM barrier of G^−^ bacteria impedes the penetration of most antimicrobial drugs, significantly reducing or even eliminating their efficacy against G^−^ pathogens [27]. Consequently, the capacity of antimicrobial drugs to traverse the OM is a vital consideration in the quest for broad-spectrum antibacterial agents. To further investigate whether LBA affects the OM permeability of *C. sakazakii*, we measured the changes in its OM permeability.

**Figure 3:**
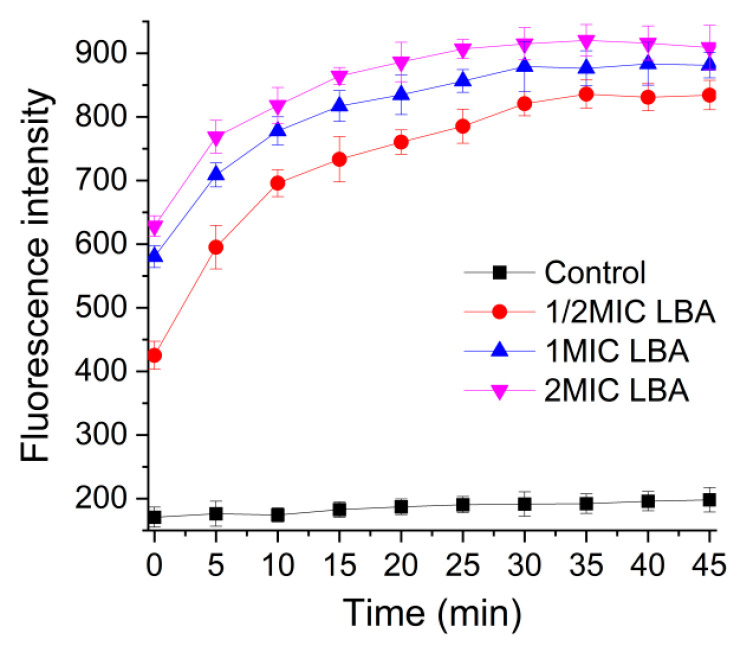
Changes in the OM permeability of *C. sakazakii* after LBA treatment.

The permeability of the OM of *C. sakazakii* was evaluated by monitoring the fluorescence level of NPN uptake. NPN, a hydrophobic fluorescence probe, is weakly fluorescent in an aqueous environment while intensely fluorescent in the hydrophobic or non-polar interiors once the cell membrane is destroyed by LBA, which causes an increase in fluorescence intensity of NPN [28]. In Figure 3, a dramatic increase up to 10 min occurs when LBA is exposed to *C. sakazakii*, then the fluorescence intensity of NPN uptake remains stable for 30 min, indicating OM permeabilization. Moreover, the fluorescence intensity was positively correlated with the concentration of LBA. The result indicated that LBA could rapidly increase the OM permeability of *C. sakazakii* in a dose-dependent manner, consistent with previous findings [20], and further strengthened by its consistency with the finding of Section 3.2 that the rise in AKP activity confirms periplasmic leakage. Previous studies reported that an increase in bacterial cell membrane permeability can cause irreversible damage to the cell membrane’s integrity, which leads to the leakage of intracellular substances, such as proteins, nucleic acids, etc., and eventually cell death [29]. We speculate that LBA may induce the leakage of intracellular materials by increasing the permeability of OM, damaging the integrity of the cell membrane, and leading to cellular death.

It is reported that the lactic acid acts against G^−^ bacteria by accessing the periplasm through the water-filled porin proteins of the OM, considering that it is a small water-soluble molecule [30]. LBA, containing eight hydroxyl groups, has a higher water solubility than lactic acid. Hence, we considered that LBA could cross the OM and follow the water-filled porin proteins to arrive in the periplasm. The OM is an essential load-bearing element in G^−^ bacteria and is even stiffer than the cell wall [31]. Lipopolysaccharide (LPS) is a main component of the peculiar lipid bilayer of the OM, which is also a barrier to prevent the entry of antibiotics and keep the vitality of most G^−^ bacteria [32,33]. Previous studies indicated that the increase in the OM permeability in G^−^ bacteria was triggered by disordering the LPS layer [34]. Therefore, we speculated that the increase in LBA on OM permeability was associated with the disruption of the LPS layer caused by LBA. Studies reported that the destruction of the OM by phenyllactic acid and lactic acid indicated that the mechanism was the disruption of molecular interactions between OM components due to the protonation of anionic components [30,35], which was similar to our results. LBA requires more time to achieve maximum absorption of NPN by *C. sakazakii* compared to phenyllactic acid (20 s); it requires less time (30 min) compared to the antimicrobial polysaccharide arginine-functionalized chitosan [36]. The phenomenon could be associated with the molecular weight of antimicrobial agents, as the molecular weight of LBA (Mw = 358.3 g/moL) is greater than that of phenyllactic acid (Mw = 166.2 g/moL), but less than that of arginine-functionalized chitosan (Mw ≈ 2000–7000 g/moL).

### 3.4. Protein Leakage and SDS-PAGE Analysis

**Figure 4:**
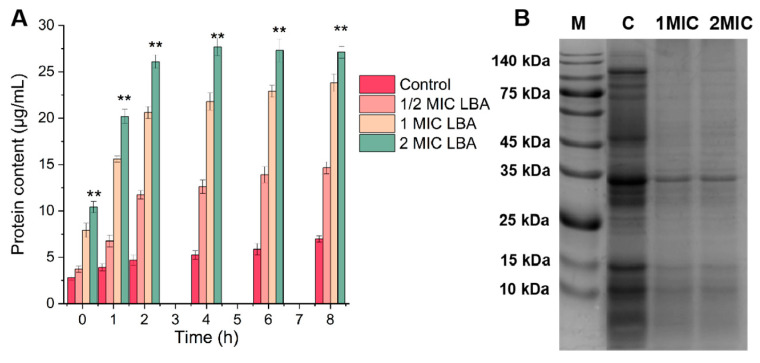
Changes in the leakage (**A**) and SDS-PAGE profile (**B**) of proteins in *C. sakazakii* after LBA treatment. Lane 1 is the marker and lanes 2–4 are the control, LBA treated with 1, and 2 MIC LBA, respectively. ‘**’, *p* < 0.01.

Protein plays a vital role in the physiological metabolism of bacteria; overleakage leads to cell death due to its impact on the normal metabolic function of *C. sakazakii* [36]. Figure 4A displays the protein leakage of *C. sakazakii*, as indicated by the protein contents in the *C. sakazakii* supernatant. During incubation, the leakage of proteins from untreated *C. sakazakii* cells showed no obvious change; the average quantity of proteins leaked was 4.91 μg/mL. However, the protein leakage of *C. sakazakii* cells significantly increased after exposure to LBA at 1/2×, 1×, and 2× MIC, and correlated positively with incubation time (*p* < 0.05). LBA at 2× MIC induced a rapid increase in protein leakage from *C. sakazakii* within an hour, with the level peaking at 27.71 μg/mL after 4 h of treatment. This trend is consistent with the cell membrane permeability results, suggesting that the decline of protein contents in *C. sakazakii* cells arises from the leakage of proteins due to increased cell membrane permeability [11]. Due to protein leakage being irreversible [37], LBA may damage the cell membrane by leaking protein in *C. sakazakii*, eventually leading to cell inactivation, confirming our previous speculation on the OM permeability results. Additionally, the decrease in bacterial protein content may stem from protein degradation or protein synthesis inhibition [22]. Therefore, the soluble proteins extracted from *C. sakazakii* were further analyzed using SDS-PAGE.

Figure 4B displays the SDS-PAGE image of *C. sakazakii* proteins exposed to different LBA concentrations. Compared with the control groups, which displayed strong and clear protein bands, *C. sakazakii* cells treated with LBA showed conspicuously faint protein bands, some of which were even undetectable. The protein bands of *C. sakazakii* were significantly reduced and partially disappeared when exposed to LBA at 1× and 2× MIC. The SDS-PAGE profiles were consistent with our previous studies in other bacteria; the protein bands in MRSA and *P. fluorescens* were weaker and even disappeared after LBA treatment [16]. The same phenomenon was also observed in *C. sakazakii* after treatment with natural extracts of olive oil polyphenols and *Houttuynia cordata* Thunb., presumably since they inhibited bacterial protein synthesis or caused marked protein leakage by damaging cellular morphology [11,38]. Another phenomenon was observed in *Yersinia enterocolitica* treated with citral; this treatment does not induce remarkable leakage of cellular fluid; rather, it synthesizes chaperones or small proteins to mitigate external stress by markedly elevating small-molecule protein levels [39]. Collectively, LBA decreased protein contents and activity in *C. sakazakii* through damaging bacterial proteins, inhibiting protein synthesis, or causing protein leakage by increasing the OM permeability, which ultimately leads to bacterial death.

### 3.5. Changes in Morphology and Ultrastructure

**Figure 5:**
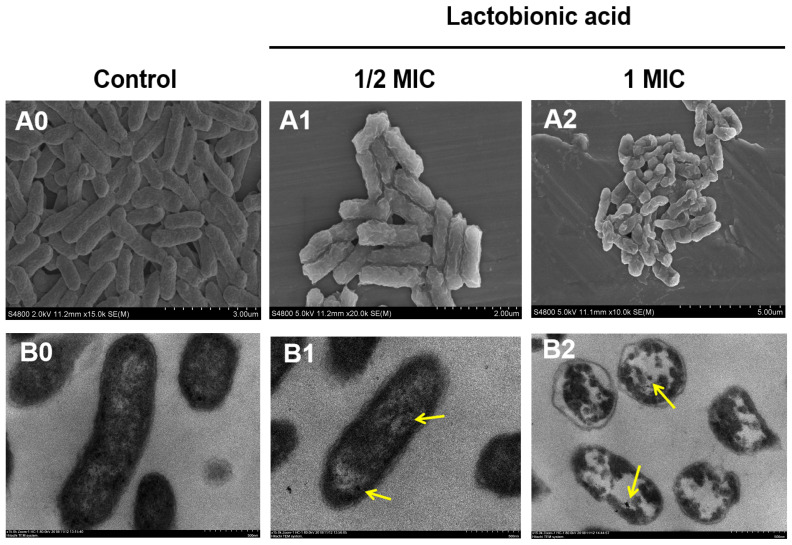
SEM images and TEM images of *C. sakazakii* cells treated with different LBA concentrations. (**A0**–**A2**) are SEM images of the control cells, cells treated with 1/2 MIC, and 1 MIC LBA, respectively; (**B0**–**B2**) are TEM images of the control cells, cells treated with 1/2 MIC, and 1 MIC LBA, respectively. Yellow arrows point to dense granules or tightly condensed substances.

The morphology and ultrastructure of *C. sakazakii* under LBA exposure were observed by SEM and TEM. The untreated cells displayed an intact rod-shaped morphology with a clear and relatively smooth surface by SEM (Figure 5(A0)), as well as a complete cell structure and a closely apposed membrane-wall interface, and cytoplasm of uniform electron density by TEM (Figure 5(B0)). While *C. sakazakii* cells treated with LBA at 1/2 MIC displayed a rough surface with marked wrinkles and compression (Figure 5(A1)), this may be caused by cell wall deformation or cytoplasm rarefaction (Figure 5(B1)). The TEM image shows that the general morphological structure of *C. sakazakii* cells was still retained after exposure to LBA at 1/2 MIC, but the partial separation of the cell wall and cell membrane was observed (Figure 5(B1)). When bacterial cells were treated with LBA at 1 MIC they were severely damaged (Figure 5(A2,B2)). Most *C. sakazakii* cells treated with 1 MIC LBA were wizened and appeared to clump with irregular and wrinkled surfaces, and showed adhesion and aggregation of damaged cells or cellular debris (Figure 5(A2)). Moreover, the TEM image showed a separation between the cell wall and membrane, internal voids, and severe plasmolysis in *C. sakazakii* cells after being treated with 1 MIC LBA; the cell wall and cell membrane were partially damaged, and massive intracellular material leaks were observed (Figure 5(B2)), indicating that the cells suffered serious damage. The images of cells treated with LBA at 2 MIC were omitted because they showed near-complete cellular disintegration indistinguishable from debris. Similar observations have been reported in our previous studies and by other researchers, that morphological and ultrastructural noticeable disruption of *P. fluorescens* [20], *V. parahaemolyticus* [21], and *Aeromonas salmonicida* [40] cells after LBA treatment, showing disruption of the cell wall and cell membrane, thinning of cytoplasm or plasmolysis, cell distortion, and leakage of intracellular materials.

The findings supported the results of the above experiments, confirming that the action mode of LBA against *C. sakazakii* is closely associated with the damage of the cell wall and membrane caused by increasing the permeability of the cell wall and membrane, leading to cell inactivation. Notably, the membrane damage observed by microscopic observation and the leakage of intracellular proteins further reinforced the evidence of OM-specific permeabilization by LBA. Additionally, some dense particles or tightly condensed substances (yellow arrows) were observed in the TEM images of the LBA-treated group. Liu et al. (2018) and Moussa et al. (2007) found similar substances in bacteria, which were assumed to be intracellular DNA denaturation and protein abnormalities caused by antimicrobial agents [41,42]. This implies to us that intracellular biopolymers (such as DNA and proteins) in cells may be another antibacterial mode of LBA against *C. sakazakii*. 

### 3.6. DNA Interaction Analysis

DNA, one of the most crucial genetic materials, controls bacterial growth, development, and genetic processes by guiding the synthesis of RNA and proteins [43]. The loss or destruction of DNA directly affects bacterial survival. In recent years, antibacterial drugs that disturb the functions of intracellular genetic materials by binding to or inhibiting DNA biosynthesis have been regarded as a common mode of antibacterial action [44,45]. Hence, the interaction between LBA and the genetic DNA of *C. sakazakii* was determined with a fluorescence probe by competitive binding.

**Figure 6:**
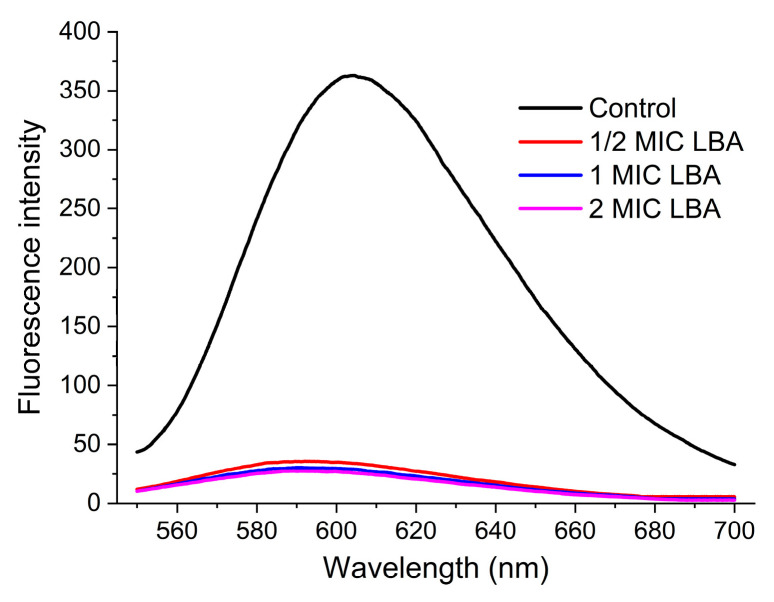
Fluorescence spectra (550–700 nm) of the EB-DNA complex upon titration with increasing LBA concentration.

Ethidium bromide (EB), as a fluorescence probe, was used in this experiment, which enhances fluorescence intensity by intercalating between adjacent base pairs of the DNA double helix; by contrast, enhanced fluorescence quenching occurs when coexisting with other molecules that have a similar binding mode to EB [46]. Figure 6 shows that significant fluorescence quenching of DNA-EB complexes was observed in the LBA-treated groups, and the quenching intensity increased slightly with the increasing concentration of LBA. The phenomenon indicated that LBA could competitively bind to DNA by intercalating into the base pairs of the DNA helix, removing EB molecules that have embedded [47]. Additionally, previous reports have shown that antimicrobial agents may interfere with the conformation and the normal functions of DNA by binding to the DNA [48]. Based on the above, we propose that LBA also exerts its antibacterial effect by binding to genomic DNA of *C. sakazakii*, thereby perturbing its conformation and function, which disrupts cell growth and viability. This proposed mode of action is consistent with findings in other bacterial species, including MRSA and *P. fluorescens*, indicating that LBA binding to genomic DNA represents a universal mechanism of action of LBA against bacteria. Other organic acids, such as Kojic acid, have also been reported to have a similar result, which could bind to the genomic DNA to affect the survival of *E. coli* biofilms [47].

## 4. Conclusions

In conclusion, LBA exerted effective antibacterial and bactericidal properties against *C. sakazakii*. The action mode of LBA against *C. sakazakii* was through destroying the integrity of the cell wall and cell membrane, as well as changing cell morphology and ultrastructure, which was associated with the increase of OM permeability, causing the leakage of intracellular proteins, leading to a decrease of the content and activity of proteins, and damaging the DNA by directly binding to it, disturbing normal cellular function, and eventually leading to cell inactivation. Hence, LBA could be a potential candidate for a natural food additive with antibacterial properties for application in the food industry.

## Data Availability

The data of this study are available from the authors upon reasonable request.
